# Persistent COVID‐19 Symptoms in General Population and Healthcare Workers: Systematic Review With Meta‐Analysis

**DOI:** 10.1002/hsr2.73456

**Published:** 2026-10-29

**Authors:** Eduarda Boufleuer, Tainara Wink Vieira, Victória Tiyoko Moraes Sakamoto, Fernando Anschau, Juliana Petri Tavares, Daiane Dal Pai

**Affiliations:** ^1^ Federal University of Rio Grande do Sul Porto Alegre Brazil; ^2^ State Health Department of Rio Grande do Sul Porto Alegre Brazil; ^3^ Grupo Hospitalar Conceição Porto Alegre Brazil; ^4^ Pontifícia Universidade Católica do Rio Grande do Sul Porto Alegre Brazil

**Keywords:** cohort studies, COVID‐19, health personnel, long COVID, post‐acute COVID‐19 syndrome, systematic review

## Abstract

**Background and Aims:**

COVID‐19 can cause persistent symptoms that continue after the infection, condition known as long COVID. This study aimed to identify the prevalence of persistent signs and symptoms of COVID‐19 in individuals with confirmed SARS‐CoV‐2 infection and among healthcare workers.

**Methods:**

This is a systematic review with meta‐analysis guided by PRISMA and registered in PROSPERO (CRD42023460632). Cohort studies with a minimum follow‐up of 12 weeks after COVID‐19 infection were included. Searches were conducted in PubMed, Scopus, Embase, Scielo, and LILACS/BVS databases and gray literature, in November 2023. Rayyan was used for article selection, and pooled prevalence meta‐analysis for each symptom was conducted in R. Methodological quality was assessed using the Newcastle‐Ottawa Scale.

**Results:**

A total of 2,456 studies were identified, with 59 articles included in the systematic review and 58 in the meta‐analysis, comprising 196,863 participants. The symptoms with the highest pooled prevalence (95% CI) were fatigue (0.29[0.24–0.36], 95% PI: 0.05–0.78) and dyspnea (0.16[0.12–0.22], 95% PI: 0.01–0.71).

**Conclusion:**

Healthcare workers showed lower prevalence rates for some persistent symptoms (fatigue, dyspnea, cough, taste changes, and gastrointestinal symptoms) than the general population. Most studies were of fair quality. Despite the findings of a high prevalence of persistent symptoms, high heterogeneity remained a key limitation.

## 1 Introduction

COVID‐19, the disease caused by the SARS‐CoV‐2 virus, has led to over 775 million cases and more than 7 million deaths globally, with Brazil accounting for approximately 10% of global fatalities [1]. In its acute phase, COVID‐19 primarily affects the respiratory system, with symptoms such as cough, dyspnea, runny nose, and sore throat [2, 3, 4]. However, symptoms can also present in gastrointestinal, musculoskeletal, neurological (e.g., headache), febrile, olfactory and gustatory dysfunctions, and cardiac manifestations [2, 5, 6].

It is well known that the severity of the disease is highly variable, with some patients experiencing mild illness and others becoming severely ill, requiring hospitalization and post‐discharge follow‐up [3]. The disease is primarily transmitted through respiratory droplets and aerosols, making mask use (as a personal protective equipment—PPE) and social distancing the most effective measures to prevent its spread [2, 7, 8].

As the pandemic progressed, it became evident that the disease could lead to long‐term systemic involvement, with persistent symptoms and sequelae, known as Post‐COVID Syndrome or Long COVID [9]. The National Institute for Health and Care Excellence (NICE) and the World Health Organization (WHO) define this syndrome as a condition in which symptoms persist for more than 3 months (12 weeks) after the acute phase of infection and cannot be explained by another diagnosis. Nonetheless, NICE acknowledges that the term Long COVID is also used to describe symptoms occurring after the infection, including the post‐acute phase (4 to 12 weeks) [10, 11].

Persistent COVID‐19 symptoms can vary and occur in different combinations or intensities. The most frequently reported symptoms include fatigue, dyspnea, muscle pain [12, 13, 14, 15], headache, loss of smell and taste, and exercise intolerance [15, 16, 17]. There is still no consensus on how long these persistent symptoms may last [11]. In this context, a detailed analysis of post‐COVID symptoms is essential to understand their diagnostic implications and develop management strategies to mitigate their impact.

It is crucial to recognize that healthcare workers have been on the frontline of the pandemic response since the first confirmed cases [18], facing numerous changes in workflow and care logistics, which have led to considerable exhaustion [19]. The widespread need for PPE among the general population contributed to shortages of these materials for healthcare workers, increasing their risk of infection due to exposure to high viral loads while caring for infected patients [18, 20, 21, 22, 23].

Given this scenario, it was hypothesized that healthcare workers might suffer more from persistent symptoms than the general population, as some studies [24, 25] indicate higher seroprevalence of infection among healthcare professionals. This study is particularly relevant due to a gap in the literature on persistent symptoms in the general population, and an additional concern regarding healthcare workers, who experienced higher exposure to both viral load and prolonged contact.

In light of these considerations, the research question was posed: “what are the most prevalent persistent signs and symptoms resulting from SARS‐CoV‐2 infection in the general population and healthcare workers?” To address this question, the aim of this study was to identify the prevalence of persistent signs and symptoms of COVID‐19 in the infected general population and among healthcare professionals.

## 2 Materials and Methods

### 2.1 Design

This study is a systematic literature review with meta‐analysis. The principles of the Preferred Reporting Items for Systematic Reviews and Meta‐Analyses (PRISMA) [26] were followed, and the relevant checklist was used for the development of the study protocol, the conduct of the study, and the drafting of the manuscript. It was registered in the International Prospective Register of Ongoing Systematic Reviews (PROSPERO) under number CRD42023460632, on September 5, 2023.

The same protocol registered in PROSPERO also supported a previously published systematic review addressing the psychological and cognitive sequelae of COVID‐19 [27]. The two reviews have distinct objectives and outcomes: psychological and cognitive outcomes in the previous review [27] and persistent physical signs and symptoms in the present analysis. Although the search strategy and eligibility criteria were shared, data extraction and meta‐analyses were conducted separately according to the different outcomes addressed in each review.

### 2.2 Search Methods

Searches were conducted in the PubMed, Scopus, Embase, Scielo, and LILACS/BVS databases, as well as through manual searches in the reference lists of selected studies and gray literature. Since this is a recent topic, no language filter was applied; the temporal filter was aligned with the timeline of the pandemic. Descriptors in Health Sciences (DeCS) and Medical Subject Headings (MeSH), such as “Post‐Acute COVID‐19 Syndrome,” “Long COVID,” and “Cohort Studies,” were used with Boolean operators OR and AND to structure the search strategies for each database. Complete search strategies are presented in Supplementary 1. Database searches were conducted in November 2023.

The PECOs strategy [28] was used, where the population (P) included the general population and healthcare workers; the exposure (E) was SARS‐CoV‐2 infection; there was no comparison group (C); the outcome (O) comprised persistent COVID‐19 signs and symptoms; and the studies (s) of interest were observational cohort studies.

### 2.3 Inclusion and Exclusion Criteria

Longitudinal observational cohort studies were included, considering the focus on prevalence meta‐analysis. Cohort studies involving individuals with confirmed SARS‐CoV‐2 infection through PCR (Polymerase Chain Reaction) or antigen tests were included, provided they assessed persistent symptoms following infection based on the period defined by WHO and NICE, as detailed in the introduction.

Studies were excluded if they included individuals diagnosed solely by clinical evaluation or those with suspected cases based on symptoms. Studies where diagnosis was based on serology and the follow‐up period after infection could not be estimated were also excluded. Exclusively pediatric populations were excluded due to the lack of a well‐defined condition in this group. Additionally, studies with follow‐up of less than 3 months post‐infection or with an unspecified follow‐up period were excluded. Articles that lacked sufficient data for symptom extraction for meta‐analysis were excluded if authors did not respond to email requests within 1 month, after two contact attempts.

### 2.4 Data Processing

The results of the article searches were imported into Endnote, where duplicates were removed. The remaining articles were uploaded to Rayyan, where two independent reviewers conducted the selection process. Disagreements were resolved by consensus, and if unresolved, a third reviewer with expertise in the topic and selection criteria made the final decision. Agreement between reviewers was assessed using kappa statistic [29], applied at the abstract screening stage.

Once article selection was finalized, data were exported to an Excel spreadsheet developed by the authors, containing key study variables. Extracted variables included study identification, sample characteristics, and follow‐up duration after COVID‐19 diagnosis. When studies reported follow‐up time in ranges, the midpoint was used to allocate the study into analysis groups, which may have resulted in some misclassification of follow‐up duration.

### 2.5 Search Outcomes

The outcomes of interest were persistent COVID‐19 signs and symptoms, initially extracted individually without data imputation. When necessary information was missing, the authors were contacted. To facilitate meta‐analysis, clinically related outcomes were grouped into predefined symptom categories (fatigue: fatigue, tiredness, weakness, reduced physical capacity, and exercise intolerance; palpitations: palpitations and tachycardia). When a study reported multiple outcomes within the same category, a single estimate was included to avoid double counting, and the most prevalent outcome was selected.

### 2.6 Data Abstraction

Meta‐analyses were performed using the “meta” package version 7.0‐0 in R software version 4.4.1, with a random‐effects model and the inverse‐variance method. A logit transformation was applied before pooling, and between‐study variance (τ^2^) was estimated using the restricted maximum‐likelihood (REML) method. A separate prevalence meta‐analysis was conducted for each persistent symptom using a 95% confidence interval. The analysis code is provided in Supplementary 2 and the full data extraction table is provided in Supplementary 3.

Heterogeneity was calculated using the Q‐test (chi‐square) and the I^2^ inconsistency index, with significance set at *p* ≤ 0.05. Analyses with I^2^ ≤ 25% were considered homogeneous; I^2^ > 25% and < 75% as moderate heterogeneity; and I^2^ ≥ 75% as high heterogeneity [30].

Given that all outcomes presented high heterogeneity, subgroup analyses were conducted based on whether participants were healthcare workers, follow‐up time post‐infection, cohort type, study location, hospitalization status, and study quality. Differences in prevalence between subgroups were considered statistically significant at *p* < 0.05.

The decision not to perform a sensitivity analysis (i.e., removing one study at a time) was based on the clinical and methodological variability across the included studies. Given the existing heterogeneity, excluding individual studies was unlikely to substantially change the overall results and was therefore not considered necessary.

### 2.7 Quality Appraisal

The methodological quality of the studies was assessed using the Newcastle–Ottawa Scale (NOS), adapted for cohort studies [31]. This scale consists of three domains: participant selection, group comparability, and outcome and follow‐up assessment. Based on the total star score, studies were classified as poor (0–3 stars), fair (4–6 stars), or good quality (7–9 stars).

To assess publication bias, Egger's [32] test and funnel plot was performed for each outcome (Supplementary 4). The certainty of the evidence was not performed, since included studies were exclusively cohorts conducted in the pandemic context, with assumed high risk of bias.

## 3 Results

Findings present the prevalence of persistent COVID‐19 signs and symptoms identified through this systematic review and meta‐analysis. As shown in Figure 1, the literature search yielded 2,456 articles related to the topic. After initial screening, 1,229 duplicates were removed. In the subsequent phase, after title and abstract screening, 203 articles were assessed in full. Of these, 141 were excluded for not meeting the inclusion/exclusion criteria or due to insufficient data extraction, four were excluded for being from the same cohort, and one was not available in full. One article was included from gray literature.

**Figure 1:**
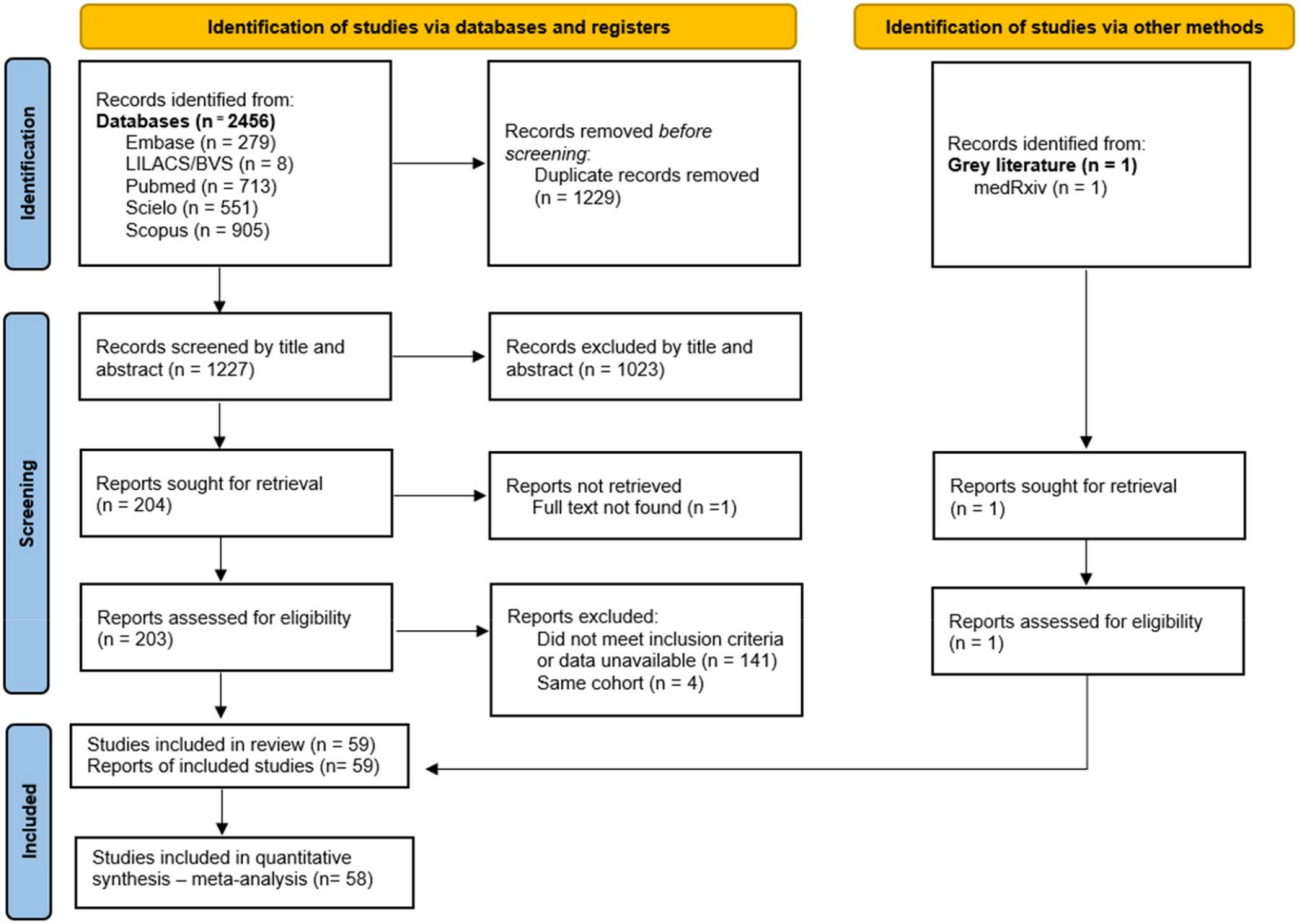
Summary of systematic review steps according to PRISMA. Porto Alegre, RS, Brazil, 2026. n = Number of studies.

In total, 59 studies were included in the systematic review of persistent COVID‐19 symptoms, of which 58 were selected for quantitative analysis. One article was excluded from the meta‐analysis because it reported a single outcome that was not among those selected for quantitative analysis [33]. The total population included in the systematic review comprised 196,863 participants. The kappa index was κ = 0.792 (*p* < 0.001), indicating substantial agreement [29]. Of the 58 articles included in the meta‐analysis, 6 [34, 35, 36, 37, 38, 39] focused exclusively on healthcare workers, and 2 [40, 41] included both healthcare workers and the general population, with stratified analyses, allowing for stratified inclusion in the meta‐analysis; thus, 8 studies involved healthcare workers.

Table 1 displays the general characteristics of the included studies, such as cohort type, basic demographic data of each population, and methodological quality based on the NOS [31]. The stratified assessment of each NOS domain is shown in Supplementary 5. Most studies were prospective cohorts (81.4%), and the quality was predominantly rated as fair (89.8%). Sample sizes varied greatly, from 27 participants in the smallest study [46] to 127,859 in the largest [69].

**Table 1:** General characteristics of the studies included in the systematic review, according to study identification, cohort characteristics, sex, mean age, follow‐up duration, sample size, and Newcastle‐Ottawa Scale (NOS) score. Porto Alegre, RS, Brazil, 2026.

Identification	Cohort	Sex (female)	Mean age	Follow‐up (months)	Sample^a^	NOS^b^
González‐Ermosillo 2021 [42]	Prospective	34.6%	51.0	6	130	4
Hossain 2021^c^ [40]	Prospective	27.6%	38.8	5	133^d^	4
					2,065^e^	
Malinowska 2021 [43]	Prospective	22.4%	53.0	6	67	4
Martinez 2021 [34]	Retrospective	75.4%	37.0	5.5	260	4
Och 2021 [44]	Prospective	50.6%	70.0	6	73	4
Scherlinger 2021 [45]	Prospective	60.0%	40.0	6	30	4
Ali 2022 [46]	Prospective	73.1%	42.8	14.5	27	6
Amorim 2022 [47]	Prospective	40.6%	48.0	3	780	4
Aparisi 2022 [48]	Prospective	64.3%	54.8	3	70	4
Asadi‐Pooya 2022 [49]	Prospective	45.3%	41.0	3	2,696	4
Buonsenso 2022 [50]	Prospective	45.7%	44.6	7.5	107	6
Carazo 2022 [35]	Retrospective	77.2%	40.0	3	1,783	5
Desgranges 2022^c^ [41]	Prospective	69.4%^d^	38.0^d^	5	281^d^	6
		48.2%^e^	51.0^e^		137^e^	
Dryden 2022 [51]	Prospective	51.3%	52.0	3	1,873	5
Estrada‐Codecido 2022 [52]	Retrospective	55.4%	38.0	3	466	4
Fang 2022 [53]	Prospective	52.1%	68.0	12	1,233	5
Fernández‐de‐Las‐Peñas 2022 [54]	Prospective	46.5%	61.0	8.4	1,969	5
Förster 2022 [55]	Retrospective	56.5%	53.0	7.8	1,459	5
Freire 2022 [56]	Prospective	46.8%	56.0	8.5	822	4
González 2022 [57]	Prospective	35.1%	61.0	12	97	4
Hastie 2022 [58]	Prospective	63.6%	44.0	12	33,281	6
Kalak 2022 [59]	Prospective	50.0%	52.1	18	166	4
Marasco 2022 [60]	Prospective	40.7%	49.9	12	435	5
Nguyen 2022 [61]	Prospective	63.8%	40.0	6	605	3
Pérez‐González 2022 [62]	Prospective	40.3%	57.0	6	248	3
Petersen 2022 [63]	Prospective	52.2%	31.3	6	170	4
Rigoni 2022 [64]	Prospective	36.1%	71.0	12	344	4
Rivera‐Izquierdo 2022 [65]	Prospective	42.6%	61.2	12	453	6
Seeßle 2022 [66]	Prospective	55.2%	57.0	12	96	4
Strahm 2022 [36]	Prospective	82.2%	38.9	4	556	7
Sun 2022 [67]	Retrospective	55.2%	62.0	15	534	4
Takakura 2022 [68]	Retrospective	52.1%	35.8	3	257	4
Tartof 2022 [69]	Retrospective	53.7%	41.2	5	127,859	7
Titze‐de‐Almeida 2022 [70]	Prospective	61.0%	41.2	6.5	236	4
Visconti 2022 [71]	Prospective	52.3%	60.5	12	88	4
Wisnivesky 2022 [72]	Prospective	64.9%	50.1	6	453	4
Zuschlag 2022 [73]	Retrospective	45.7%	62.0	12	162	4
Ballouz 2023 [74]	Prospective	49.6%	50.0	6	504	7
Bamps 2023^f^ [33]	Prospective	55.5%	55.0	12	474	4
Benoit‐Piau 2023 [75]	Prospective	61.3%	46.6	6	1,349	5
Brunvoll 2023 [76]	Prospective	71.3%	48.0	9	1,420	7
Cecchetto 2023 [77]	Retrospective	39.1%	65.0	5	225	4
D'Ávila 2023 [37]	Prospective	80.6%	42.2	6	174	4
Gentilotti 2023 [78]	Prospective	43.4%	57.2	12	1,796	5
Grewal 2023 [79]	Prospective	38.2%	63.0	12	76	4
Hyassat 2023 [38]	Prospective	70.7%	34.4	12	140	4
Klinkhammer 2023 [80]	Prospective	30.2%	64.0	9	205	4
Krishnadath 2023 [81]	Prospective	62.3%	49.0	3.5	106	4
Lemhöfer 2023 [82]	Prospective	66.4%	49.5	9.6	952	4
Magel 2023 [83]	Prospective	36.4%	61.1	6	79	4
Monteiro 2023 [84]	Prospective	69.8%	41.4	9	48	4
Oliveira 2023 [85]	Retrospective	59.2%	46.0	3	284	4
Peghin 2023 [86]	Prospective	53.5%	54.7	24	230	4
Ranucci 2023 [87]	Prospective	33.9%	63.1	17	121	4
Serrano 2023 [88]	Prospective	29.6%	62.0	12	135	4
Silva 2023 [39]	Prospective	63.9%	38.4	12	69	4
Smith 2023 [89]	Prospective	64.6%	43.1	3	5,727	5
Steinmetz 2023 [90]	Prospective	78.5%	48.2	6	104	4
Sykes 2023 [91]	Retrospective	37.5%	62.0	3	144	4

**Follow‐up (months)** = Follow‐up after COVID‐19 in months.

^a^ **N sample** = sample size of each study.

^b^ **NOS** = Newcastle–Ottawa Scale, 0–3: poor; 4–6: fair; 7–9: good.

^c^ Studies with general population and healthcare workers with stratified analyses.

^d^ data from healthcare workers.

^e^ data from the general population.

^f^ Study/outcome not included in the meta‐analysis.

Further details, including country of origin, participant comorbidities, and symptoms assessed for meta‐analysis, are provided in Supplementary 6. The most common comorbidities across studies were diabetes mellitus, hypertension, cardiac, pulmonary, renal, hepatic, and psychiatric conditions, as well as overweight and obesity. Mean age in general population ranged from 31.3 to 71 years, and among healthcare workers from 34.4 to 42.2 years. Follow‐up periods ranged from 3 to 24 months post‐diagnosis. Geographically, most studies originated from Europe (32), followed by South and North America (9 each), Asia (7), Africa (1), and one multi‐country study across Europe and South America.

Given the large number of analyzed outcomes, articles, and high heterogeneity, only the forest plot for the most prevalent symptom (fatigue) is shown in the manuscript (Figure 2). The other forest plots are presented in Supplementary 7. All data are comprehensively presented through descriptive analyses and tables summarizing outcome information.

**Figure 2:**
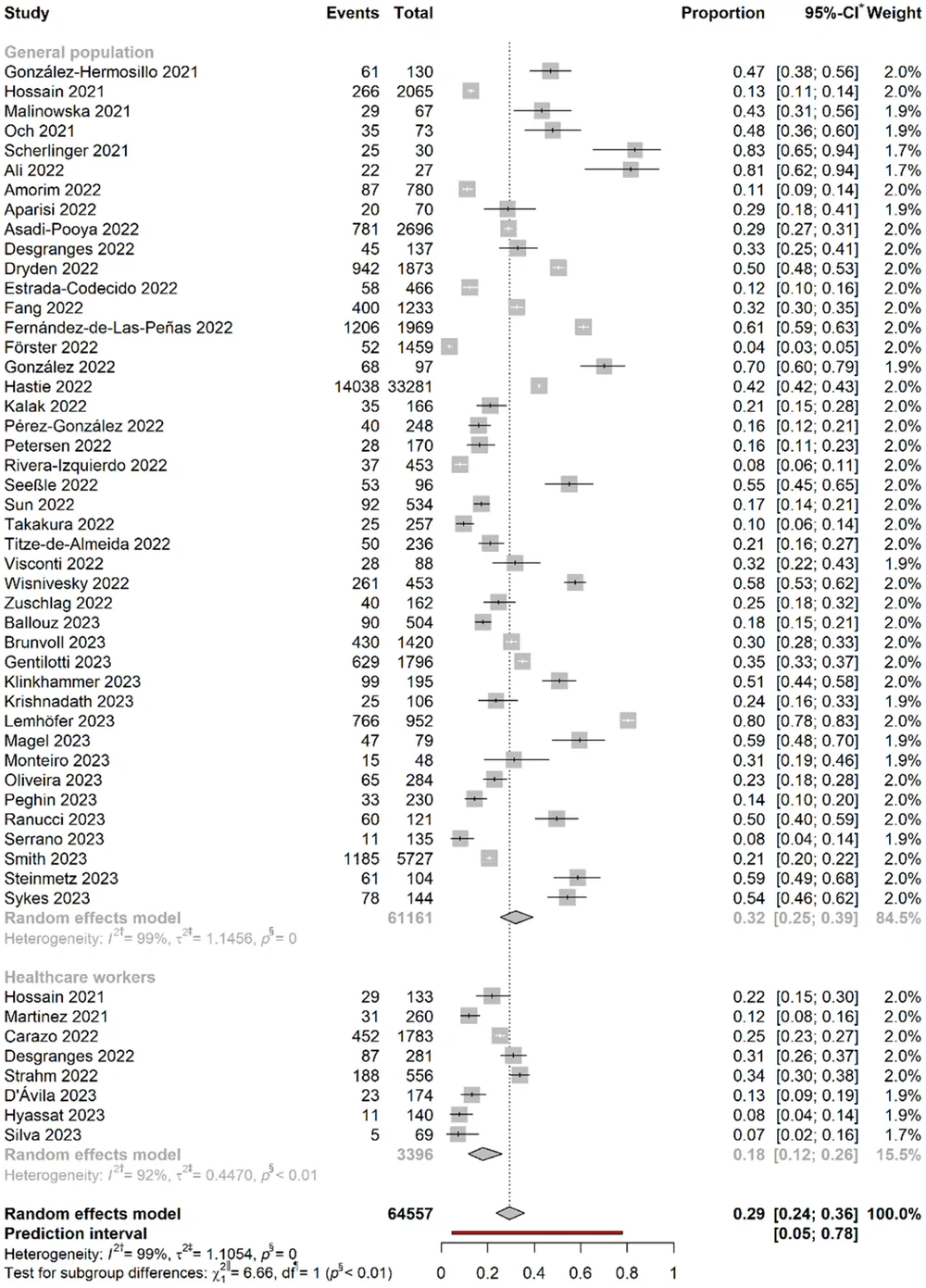
Forest plot of the most prevalent persistent symptom (fatigue). Porto Alegre, RS, Brazil, 2026.

The pooled prevalence estimates (95% CI) from the meta‐analysis are presented in descending order in Table 2, alongside prediction intervals and heterogeneity metrics. The most prevalent outcomes were fatigue (0.29 [95% CI: 0.24; 0.36], 95% PI: 0.05–0.78, I^2^ = 98.9%), dyspnea (0.16 [95% CI: 0.12; 0.22], 95% PI: 0.01–0.71, I^2^ = 99.5%), arthralgia (0.12 [95% CI: 0.07; 0.19], 95% PI: 0.01–0.72, I^2^ = 97.8%), myalgia (0.11 [95% CI: 0.07; 0.16], 95% PI: 0.01–0.59, I^2^ = 98.4%), headache (0.10 [95% CI: 0.07; 0.13], 95% PI: 0.01–0.53, I^2^ = 99.5%), hair loss (0.09 [95% CI: 0.06; 0.13], 95% PI: 0.01–0.42, I^2^ = 98.7%), olfactory dysfunction (0.08 [95% CI: 0.06; 0.12], 95% PI: 0.01–0.45, I^2^ = 97.2%), and palpitations (0.08 [95% CI: 0.05; 0.12], 95% PI: 0.01–0.49, I^2^ = 98.4%). Lower prevalence outcomes include cough, dizziness/fainting, chest pain/pressure, taste changes, gastrointestinal symptoms, skin alterations, and fever/shivering. The articles that reported each symptom are listed in Supplementary 8.

**Table 2:** Prevalence of persistent signs and symptoms: overall, general population, and healthcare workers. Porto Alegre, RS, Brazil, 2026.

General data						General population		Healthcare workers		*p* value^f^ between groups
Outcome	N data.^a^	E^b^/O^c^	General prevalence (CI 95%^d^)	Prediction (CI 95%^d^)	I^2^ ^e^/*p* value^f^	Prevalence (CI 95%^d^)	I^2^ e	Prevalence (CI 95%^d^)	I^2^ e	
Fatigue^g^	51	23,244/64,557	0.29[0.24;0.36]	[0.05;0.78]	98.9% *p* < 0.0001	0.32[0.25;0.39]	99.0%	0.18[0.12;0.26]	92.4%	0.0098
Dyspnea	47	18,125/191,101	0.16[0.12;0.22]	[0.01;0.71]	99.5% *p* < 0.0001	0.19[0.14;0.25]	99.6%	0.05[0.02;0.13]	93.9%	0.0065
Arthralgia	20	7,224/46,508	0.12[0.07;0.19]	[0.01;0.72]	97.8% *p* < 0.0001	0.12[0.07;0.21]	97.9%	0.06[0.00;0.44]	96.1%	0.5311
Myalgia	27	9,709/51,816	0.11[0.07;0.16]	[0.01;0.59]	98.4% *p* < 0.0001	0.12[0.08;0.18]	98.5%	0.04[0.01;0.16]	92.4%	0.1285
Headache	38	17,992/183,056	0.10[0.07;0.13]	[0.01;0.53]	99.5% *p* < 0.0001	0.11[0.07;0.15]	99.6%	0.06[0.03;0.11]	90.2%	0.1283
Hair loss	20	2,745/133,747	0.09[0.06;0.13]	[0.01;0.42]	98.7% *p* < 0.0001	0.08[0.05;0.13]	98.4%	0.11[0.05;0.23]	90.3%	0.5636
Olfactory dysfunction	30	4,407/48,980	0.08[0.06;0.12]	[0.01;0.45]	97.2% *p* < 0.0001	0.08[0.06;0.12]	97.4%	0.06[0.04;0.11]	5.3%	0.4426
Palpitations^h^	24	8,966/175,062	0.08[0.05;0.12]	[0.01;0.49]	98.4% *p* < 0.0001	0.09[0.05;0.13]	98.4%	0.04[0.00;0.26]	94.5%	0.4462
Cough	37	13,375/186,337	0.07[0.05;0.10]	[0.01;0.44]	98.8% *p* < 0.0001	0.08[0.05;0.12]	99.1%	0.03[0.02;0.06]	81.3%	0.0218
Dizziness/fainting	23	2,414/49,548	0.06[0.04;0.11]	[0.01;0.51]	96.7% *p* < 0.0001	0.07[0.04;0.12]	96.9%	0.04[0.01;0.17]	90.7%	0.5469
Chest pain	36	3,480/57,658	0.06[0.04;0.09]	[0.01;0.45]	96.8% *p* < 0.0001	0.07[0.04;0.11]	97.2%	0.04[0.02;0.06]	79.1%	0.0805
Taste changes	28	3,389/47,705	0.06[0.04;0.08]	[0.01;0.37]	95.4% *p* < 0.0001	0.06[0.04;0.09]	95.8%	0.01[0.01;0.03]	0.0%	0.0031
Gastrointestinal symptoms	40	6,967/186,842	0.05[0.03;0.07]	[0.00;0.37]	98.8% *p* < 0.0001	0.05[0.04;0.08]	98.9%	0.01[0.01;0.02]	0.0%	< 0.001
Skin alterations	21	6,217/178,090	0.03[0.02;0.04]	[0.00;0.16]	96.2% *p* < 0.0001	0.03[0.02;0.05]	96.4%	0.00[0.00;0.05]	**‐** i	0.1279
Fever/shivering	17	167/9,198	0.01[0.01;0.03]	[0.00;0.21]	86.6% *p* < 0.0001	0.02[0.01;0.03]	87.2%	0.00[0.00;0.05]	**‐** i	0.3079

^a^ N data.=Number of independent datasets included in the meta‐analysis; some studies (Hossain et al. and Desgranges et al.) contributed more than one dataset because results were reported separately for different populations.

^b^ E= Events.

^c^ O= Observations.

^d^ CI 95% = 95% Confidence interval.

^e^ I^2^= Higgins inconsistency Index (heterogeneity).

^f^ p‐value=Significance level.

^g^ Fatigue= fatigue, tiredness, weakness, reduced physical capacity and exercise intolerance.

^h^ Palpitations= palpitations, tachycardia.

^i^ Skin alterations and Fever/shivering do not present heterogeneity in healthcare workers because as they are composed of only one article.

Only the 15most prevalent symptoms analyzed quantitatively are presented. Additional symptoms were identified during full‐text analysis, such as vision changes [46, 51, 54, 57, 58, 65, 67, 74, 78, 85, 86, 87, 89], hearing alterations [41, 46, 51, 52, 57, 58, 64, 74, 84, 85, 87, 89], seizures [51, 78], thromboembolic events [33, 65, 67, 69], sweating pattern changes/temperature intolerance [42, 53], extremity issues like numbness or edema [38, 41, 42, 45, 46, 48, 53, 57, 64, 74, 80, 87, 89], and gait imbalance [35, 41, 42, 58, 64, 67, 78, 89].

Additional reports included weight loss/anorexia [34, 36, 40, 43, 44, 51, 52, 53, 57, 58, 67, 70, 73, 78, 89], urinary and sexual dysfunction [42, 50, 65, 84], and bleeding/hemorrhage [50, 51, 52, 78]. Respiratory symptoms like mucosal changes [55], rhinorrhea or sputum [35, 39, 51, 52, 53, 55, 58, 63, 70, 72, 78, 90], sore throat, hoarseness, or wheezing [35, 39, 52, 53, 54, 55, 57, 63, 66, 67, 70, 72, 78, 84, 91] were also noted.

Subgroup analyses addressing high heterogeneity are fully presented in Supplementary 9. Subgroups with low heterogeneity were mostly composed of few studies, which may limit representativeness.

No statistically significant differences were found in symptom prevalence by follow‐up time. Geographic location showed differences for most outcomes, except palpitations and GI symptoms. However, this may be influenced by the concentration of studies from Europe, South, and North America.

Prospective studies showed higher prevalence for symptoms such as fatigue, headache, palpitations, cough, and GI symptoms, compared to retrospective ones. Study quality was associated with differences in fatigue, myalgia, headache, cough, and GI symptoms.

Hospitalization status was significantly associated with differences in four symptoms: dyspnea, cough, palpitations, and dizziness/fainting. Cough was more prevalent among hospitalized individuals.

Subgroup analyses identified statistically significant differences (*p* < 0.05) for fatigue, dyspnea, cough, taste and GI symptoms, with lower pooled prevalences among healthcare workers. Notably, heterogeneity for taste and GI symptoms in healthcare workers was reduced (I^2^ < 25%).

## 4 Discussion

The results of this systematic review reveal that individuals with confirmed SARS‐CoV‐2 infection experienced persistent symptoms after the resolution of the acute illness, which may impact their quality of life, daily activities, return to work, and place additional strain on the healthcare system. The wide prediction intervals observed are consistent with the high heterogeneity across studies; thus, findings should be interpreted cautiously. This heterogeneity may reflect differences in study populations, hospitalization status, follow‐up duration, circulating SARS‐CoV‐2 variants, vaccination status, symptom ascertainment methods, and geographic variability. Differences in data collection methods and reliance on self‐reported symptoms may also have contributed to this variability and affected study quality, as assessed using the NOS [31]. Although subgroup analyses provided some insight into potential sources of heterogeneity, residual heterogeneity remained for several outcomes. Therefore, pooled prevalence estimates should be interpreted as overall estimates across diverse populations and may have limited generalizability to specific settings. This discussion focuses on the symptoms analyzed quantitatively.

Fatigue, identified as the most prevalent symptom (pooled prevalence of 0.29), is characterized by a sensation of tiredness and lack of energy, typically assessed through self‐report [92, 93]. A previous systematic review [94] on fatigue reported a similar prevalence (0.22), although differences may stem from the inclusion of cross‐sectional studies. As in the present review, no significant differences were found regarding follow‐up duration or hospitalization status. Persistent fatigue may involve immune dysregulation and altered cellular energy metabolism, including impaired mitochondrial function, although the contribution of these mechanisms remains incompletely understood [95]. High heterogeneity persisted even after subgroup analyses. Another study [96] conducted with individuals post‐acute COVID‐19 found higher levels of fatigue in those with other ongoing symptoms and linked fatigue to a reduced quality of life.

Respiratory symptoms, such as prolonged dyspnea and cough, are of clinical concern. Individuals with persistent respiratory symptoms may experience reduced quality of life and functional limitations [97, 98]. These symptoms may result from residual pulmonary damage or inflammation caused by the virus [99]. Persistent dyspnea may also be associated with immune dysregulation and endothelial or microvascular dysfunction, which may contribute to impaired pulmonary diffusion capacity. However, the mechanisms underlying this symptom remain heterogeneous and incompletely understood [100].

A population‐based study in Germany [17] reported post‐COVID symptom prevalence six to twelve months after infection, similar to findings from this review, including musculoskeletal symptoms like myalgia and arthralgia. These symptoms likely reflect lingering inflammation and may be even more prevalent post‐infection than during the acute phase. Another review [98] reported prevalence rates of 18% for myalgia and 33% for arthralgia.

During the acute phase of COVID‐19, olfactory and gustatory dysfunctions were commonly observed and often served as diagnostic indicators [101]. However, these symptoms are not universal across all virus variants. A Brazilian study [102] found that olfactory and taste changes during the acute phase were associated with their persistence 6–11 months post‐infection and linked to cognitive impairments. Previous reviews [16, 98] and Brazilian study [103] reported similar prevalence, supporting the present findings. However, other studies [17, 104] observed higher rates. Variability may be due to differences in definitions—some studies included any sensory change, while others focused only on complete loss of smell or taste.

Dermatological issues, such as rashes and hair loss, were also frequent, particularly among women [98, 105]. Literature on post‐infection hair loss presents inconsistent results, aligning with the wide prediction interval in this review. A German study [17] reported a similar prevalence to that found here, whereas others [106, 107] found higher rates. Variability may arise from the present review's inclusion of various hair loss patterns without stratification. Moreover, no sex‐stratified analysis was conducted, although women are more likely to perceive and report such symptoms.

Headache, the fifth most common symptom in this review, may occur in individuals with or without a prior history of headaches, which helps explain the wide range in prevalence [17, 108]. A previous review [109] suggested that headaches were more frequently reported among healthcare workers, possibly due to greater awareness and reporting. However, in this review, healthcare workers reported lower headache prevalence compared to the general population. Dizziness was also commonly reported during the acute phase, but appeared less frequent post‐infection, showing a tendency to improve over time [110]. These findings align with the low prevalence of dizziness found in this review.

Cardiovascular symptoms such as chest pain or tightness and palpitations were observed both during and after infection [17, 97, 111, 112]. A prior review [98] reported a chest pain prevalence similar to that found here, though studies vary widely. Embolism [33, 65, 67, 69], mentioned in this review, is considered a possible sequela of infection, but was not included in the meta‐analysis. One study [113] emphasized that a generalized association between COVID‐19 and increased thromboembolism risk cannot yet be confirmed.

Gastrointestinal symptoms (e.g., nausea, vomiting, diarrhea) and symptoms like tremors and fever were commonly reported during the acute phase and may persist thereafter. However, one study [114] noted uncertainty about when the GI tract is most affected during infection, and the mechanisms underlying these symptoms remain unclear. Consistent with this, although these symptoms were among the fifteen most prevalent in this review, GI symptoms and tremors/fever had low prevalence (0.05 and 0.01, respectively). Another study [17] argued that these symptoms, while reported by some, are not central features of post‐COVID syndrome.

Regarding subgroup analyses, differences by cohort type may reflect better symptom detection in prospective studies, as participants are monitored over time and may be more likely to report symptoms. Comparisons by methodological quality were limited, as most studies were of fair quality [31]. Similarly, analysis by geographic location was affected by the overrepresentation of European studies, consistent with the fact that European countries were among the first affected by COVID‐19 [115].

It was expected that symptom prevalence would decline over time post‐diagnosis [116], but no significant differences were found across time intervals. Hospitalization analysis was limited by articles classified as “some” (mixed hospitalized and non‐hospitalized populations) or “missing” (no hospitalization data), making pattern identification difficult.

The presence of funnel plot asymmetry was suggested for fatigue, myalgia, hair loss, and fever/shivering. Dyspnea showed a borderline result, suggesting a possible tendency toward asymmetry. These findings indicate that the pooled prevalence estimates for some symptoms may be influenced by reporting biases, and the estimates should therefore be interpreted with caution, as studies with smaller samples may contribute disproportionately to the observed prevalence. However, in meta‐analyses of proportions, funnel plot asymmetry may also arise from the intrinsic mean–variance relationship rather than publication bias alone. No statistical evidence of asymmetry was observed for the remaining outcomes. However, these results should be interpreted with caution, as Egger's test may be affected by substantial between‐study heterogeneity.

Stratified analysis of healthcare workers was considered essential given their high exposure, which might place them at higher risk for prolonged symptoms [18, 22]. Some symptoms showed significant differences ‐ fatigue, dyspnea, cough, taste, and GI symptoms were all less prevalent among healthcare workers. Nevertheless, these findings should be considered exploratory, given the substantial heterogeneity across studies and the limited evidence available for some healthcare‐worker subgroups.

Although the reasons for these differences could not be evaluated in the present review, they may reflect factors such as priority access to vaccination, supported by studies showing that vaccination may reduce disease severity [117], as well as their younger average age compared to the general population. Additionally, symptoms may have been underreported by healthcare workers, who, having cared for severely ill patients, may perceive their own symptoms as less severe, or may have received early treatment due to prompt symptom recognition, thus preventing or mitigating post‐acute effects. Therefore, these explanations should be interpreted as hypotheses rather than causal inferences.

## 5 Strengths and Limitations

The study is relevant due to its simultaneous analysis of multiple outcomes (15 symptoms) which enables the identification of those with the highest prevalence, as well as the breadth and robustness of its sample.

A major limitation is the reliance on self‐reported symptoms, which, even when collected through standardized questionnaires or clinical follow‐up, depend on participants’ subjective perception, potentially leading to under‐ or overestimation. Additionally, many studies included hospitalized or ambulatory patients, who tend to be more symptomatic in the acute phase and may have higher post‐infection symptom prevalence. Consequently, asymptomatic or mildly symptomatic individuals may be underrepresented, as few studies included uninfected comparison groups.

Another limitation is the potential for bias arising from methodological and population differences across studies conducted during the pandemic, including variations in study design, sample size, symptom assessment, and lack of standardized data collection methods. These differences may have contributed to the high heterogeneity observed across outcomes. In addition, the evidence of small‐study effects for some outcomes raises the possibility that publication bias may have influenced the pooled prevalence estimates. Therefore, these methodological limitations and potential biases should be considered when interpreting the pooled prevalence estimates.

Moreover, symptoms may be influenced by COVID‐19 vaccination and virus variants, which produce different symptoms and severity levels. These variables were not controlled in this review due to lack of information in most included studies and unequal global vaccination and variant distribution.

Furthermore, the omission of more recent evidence is considered a potential limitation, given the time elapsed between the literature search and the synthesis of the present manuscript. Although a leave‐one‐out analysis was not performed, the meta‐analyses used a random‐effects model, which considers both within‐ and between‐study variability when estimating study weights. Nevertheless, given the substantial variation in sample sizes across studies, the absence of a formal influence analysis represents an additional limitation that should be considered when interpreting the pooled estimates. The certainty of the evidence was not assessed using the GRADE approach, given the substantial heterogeneity and methodological differences across the included studies, which represents a limitation of this review. These limitations restrict the generalizability of the findings.

## 6 Conclusion

This systematic review explored the prevalence of post‐COVID symptoms, offering a comprehensive view of the diverse ways in which these symptoms manifest across affected populations. The significance of this study lies in its integrated approach, applying meta‐analysis to multiple outcomes to identify the most commonly reported symptoms, many of which have been addressed individually or in more limited analyses in previous research.

Findings indicate that, although symptoms such as fatigue and dyspnea are widely reported, their prevalence varies substantially among the included studies. This is evidenced by the high levels of heterogeneity (which were not substantially reduced in most subgroup analyses) and wide prediction intervals. The variability found can be partly attributed to the pandemic context in which these studies were conducted, which affected data collection methods and population characteristics.

Healthcare workers presented lower prevalence rates of persistent symptoms compared to the general population. This may be due to several factors discussed earlier, such as prioritized access to vaccination, younger average age, and possibly greater symptom resilience or earlier treatment. However, these results must be interpreted with caution in light of the existing limitations.

This consolidated perspective on symptom persistence aims not only to present prevalence estimates but also to support the development of health management strategies and promote further research. Such research will be essential for improving our understanding of the complexity and variability of post‐COVID symptoms, thereby enhancing care and support strategies for affected populations. Moreover, this review remains highly relevant given the ongoing impact of the pandemic on global health systems and the pressing need for evidence‐based public health interventions.

## Author Contributions

**Eduarda Boufleuer:** conceptualization, data curation, formal analysis, investigation, methodology, project administration, software, supervision, validation, visualization, writing – original draft, writing – review and editing. **Tainara Wink Vieira:** conceptualization, data curation, investigation, validation, visualization, writing – original draft, writing – review and editing. **Victória Tiyoko Moraes Sakamoto:** conceptualization, validation, visualization, writing – review and editing. **Fernando Anschau:** conceptualization, formal analysis, investigation, methodology, project administration, validation, visualization, writing – review and editing. **Juliana Petri Tavares:** validation, visualization, writing – review and editing. **Daiane Dal Pai:** conceptualization, data curation, formal analysis, funding acquisition, investigation, methodology, project administration, supervision, validation, visualization, writing – original draft, writing – review and editing.

## Ethics Statement

The authors have nothing to report.

## Conflicts of Interest

The authors declare no conflicts of interest.

## Transparency Statement

The lead author Eduarda Boufleuer affirms that this manuscript is an honest, accurate, and transparent account of the study being reported; that no important aspects of the study have been omitted; and that any discrepancies from the study as planned (and, if relevant, registered) have been explained. All authors have read and approved the final version of the manuscript. Eduarda Boufleuer had full access to all of the data in this study and takes complete responsibility for the integrity of the data and the accuracy of the data analysis.

## Supporting information


Supporting File 1

Supporting File 2

Supporting File 3

Supporting File 4

Supporting File 5

Supporting File 6

Supporting File 7

Supporting File 8

Supporting File 9

## Data Availability

The data that supports the findings of this study are available in the supporting material of this article.
