# Supplementary material for: Persistent COVID‐19 Symptoms in General Population and Healthcare Workers: Systematic Review With Meta‐Analysis
Source: Health Sci Rep. 2026 Oct 29;9(11):e73456. doi: 10.1002/hsr2.73456 (PMC13720233; doi:10.1002/hsr2.73456)
Supplement: Supplementary file 1 [file HSR2-9-e73456-s003.pdf]

Supplementary 2 - Analysis code software R. Porto Alegre, RS, Brazil, 2026.

```
# ============================================================
# R CODE FOR META-ANALYSIS
# ============================================================

# Packages
library(readxl)
library(meta)
library(metafor)
library(ggplot2)

# ============================================================
# DATA IMPORT
# ============================================================

BANCO <- read_excel("Supplementary 3.xlsx")


# ============================================================
# FATIGUE
# ============================================================

b_fad <- subset(BANCO, !is.na(fad) & tepoultimo == 1)

m_fad <- metaprop(
  event = fad,
  n = fadt,
  studlab = autorano,
  data = b_fad,
  method = "Inverse",
  common = FALSE,
  prediction = TRUE,
  sortvar = b_fad$ano,
  print.pval = TRUE,
  digits.pval = 3
)

m_fad

tiff(
  "m_fad.tiff",
  res = 300,
  width = 30,
  height = 40,
  units = "cm",
  compression = "lzw+p"
)

meta::forest(m_fad)

dev.off()


# Funnel plot
meta::funnel(
  m_fad,
  studlab = FALSE,
  main = "Funnel Plot Fatigue"
)

# Egger's test
metabias(
  m_fad,
  method.bias = "linreg"
)


# ============================================================
# HEADACHE
# ============================================================
```

```r
b_cef <- subset(BANCO, !is.na(cef) & tepoultimo == 1)

m_cef <- metaprop(
  event = cef,
  n = ceft,
  studlab = autorano,
  data = b_cef,
  method = "Inverse",
  common = FALSE,
  prediction = TRUE,
  sortvar = b_cef$ano,
  print.pval = TRUE,
  digits.pval = 3
)

m_cef

tiff(
  "m_cef.tiff",
  res = 300,
  width = 30,
  height = 40,
  units = "cm",
  compression = "lzw+p"
)

meta::forest(m_cef)

dev.off()


# ==============================================================
# MYALGIA
# ==============================================================

b_mial <- subset(BANCO, !is.na(mial) & tepoultimo == 1)

m_mial <- metaprop(
  event = mial,
  n = mialt,
  studlab = autorano,
  data = b_mial,
  method = "Inverse",
  common = FALSE,
  prediction = TRUE,
  sortvar = b_mial$ano,
  print.pval = TRUE,
  digits.pval = 3
)

m_mial

tiff(
  "m_mial.tiff",
  res = 300,
  width = 30,
  height = 40,
  units = "cm",
  compression = "lzw+p"
)

meta::forest(m_mial)

dev.off()


# ==============================================================
# ARTHRALGIA
```

```
# ============================================================

b_artral <- subset(BANCO, !is.na(artral) & tepoultimo == 1)

m_artral <- metaprop(
  event = artral,
  n = atralt,
  studlab = autorano,
  data = b_artral,
  method = "Inverse",
  common = FALSE,
  prediction = TRUE,
  sortvar = b_artral$ano,
  print.pval = TRUE,
  digits.pval = 3
)

m_artral

tiff(
  "m_artral.tiff",
  res = 300,
  width = 30,
  height = 40,
  units = "cm",
  compression = "lzw+p"
)

meta::forest(m_artral)

dev.off()


# ============================================================
# FEVER/SHIVERING
# ============================================================

b_febtrem <- subset(BANCO, !is.na(febtrem) & tepoultimo == 1)

m_febtrem <- metaprop(
  event = febtrem,
  n = febtremt,
  studlab = autorano,
  data = b_febtrem,
  method = "Inverse",
  common = FALSE,
  prediction = TRUE,
  sortvar = b_febtrem$ano,
  print.pval = TRUE,
  digits.pval = 3
)

m_febtrem

tiff(
  "m_febtrem.tiff",
  res = 300,
  width = 30,
  height = 40,
  units = "cm",
  compression = "lzw+p"
)

meta::forest(m_febtrem)

dev.off()


# ============================================================
```

```
# HAIR LOSS
# ==============================================================

b_cabelo <- subset(BANCO, !is.na(cabelo) & tepoultimo == 1)

m_cabelo <- metaprop(
  event = cabelo,
  n = cabelot,
  studlab = autorano,
  data = b_cabelo,
  method = "Inverse",
  common = FALSE,
  prediction = TRUE,
  sortvar = b_cabelo$ano,
  print.pval = TRUE,
  digits.pval = 3
)

m_cabelo

tiff(
  "m_cabelo.tiff",
  res = 300,
  width = 30,
  height = 40,
  units = "cm",
  compression = "lzw+p"
)

meta::forest(m_cabelo)

dev.off()


# ==============================================================
# SKIN ALTERATIONS
# ==============================================================

b_pele <- subset(BANCO, !is.na(pele) & tepoultimo == 1)

m_pele <- metaprop(
  event = pele,
  n = pelet,
  studlab = autorano,
  data = b_pele,
  method = "Inverse",
  common = FALSE,
  prediction = TRUE,
  sortvar = b_pele$ano,
  print.pval = TRUE,
  digits.pval = 3
)

m_pele

tiff(
  "m_pele.tiff",
  res = 300,
  width = 30,
  height = 40,
  units = "cm",
  compression = "lzw+p"
)

meta::forest(m_pele)

dev.off()
```

```r
# ==============================================================
# OLFACTORY DYSFUNCTION
# ==============================================================

b_olfa <- subset(BANCO, !is.na(olfa) & tepoultimo == 1)

m_olfa <- metaprop(
  event = olfa,
  n = olfat,
  studlab = autorano,
  data = b_olfa,
  method = "Inverse",
  common = FALSE,
  prediction = TRUE,
  sortvar = b_olfa$ano,
  print.pval = TRUE,
  digits.pval = 3
)

m_olfa

tiff(
  "m_olfa.tiff",
  res = 300,
  width = 30,
  height = 40,
  units = "cm",
  compression = "lzw+p"
)

meta::forest(m_olfa)

dev.off()


# ==============================================================
# TASTE CHANGES
# ==============================================================

b_palad <- subset(BANCO, !is.na(palad) & tepoultimo == 1)

m_palad <- metaprop(
  event = palad,
  n = paladt,
  studlab = autorano,
  data = b_palad,
  method = "Inverse",
  common = FALSE,
  prediction = TRUE,
  sortvar = b_palad$ano,
  print.pval = TRUE,
  digits.pval = 3
)

m_palad

tiff(
  "m_palad.tiff",
  res = 300,
  width = 30,
  height = 40,
  units = "cm",
  compression = "lzw+p"
)

meta::forest(m_palad)

dev.off()
```

```r
# ============================================================
# DIZZINESS/FAINTING
# ============================================================

b_tondesm <- subset(BANCO, !is.na(tondesm) & tepoultimo == 1)

m_tondesm <- metaprop(
  event = tondesm,
  n = tondesmt,
  studlab = autorano,
  data = b_tondesm,
  method = "Inverse",
  common = FALSE,
  prediction = TRUE,
  sortvar = b_tondesm$ano,
  print.pval = TRUE,
  digits.pval = 3
)

m_tondesm

tiff(
  "m_tondesm.tiff",
  res = 300,
  width = 30,
  height = 40,
  units = "cm",
  compression = "lzw+p"
)

meta::forest(m_tondesm)

dev.off()


# ============================================================
# DYSPNEA
# ============================================================

b_dispn <- subset(BANCO, !is.na(dispn) & tepoultimo == 1)

m_dispn <- metaprop(
  event = dispn,
  n = dispnt,
  studlab = autorano,
  data = b_dispn,
  method = "Inverse",
  common = FALSE,
  prediction = TRUE,
  sortvar = b_dispn$ano,
  print.pval = TRUE,
  digits.pval = 3
)

m_dispn

tiff(
  "m_dispn.tiff",
  res = 300,
  width = 30,
  height = 40,
  units = "cm",
  compression = "lzw+p"
)

meta::forest(m_dispn)

dev.off()
```

```
# ============================================================
# COUGH
# ============================================================

b_tosse <- subset(BANCO, !is.na(tosse) & tepoultimo == 1)

m_tosse <- metaprop(
  event = tosse,
  n = tosset,
  studlab = autorano,
  data = b_tosse,
  method = "Inverse",
  common = FALSE,
  prediction = TRUE,
  sortvar = b_tosse$ano,
  print.pval = TRUE,
  digits.pval = 3
)

m_tosse

tiff(
  "m_tosse.tiff",
  res = 300,
  width = 30,
  height = 40,
  units = "cm",
  compression = "lzw+p"
)

meta::forest(m_tosse)

dev.off()


# ============================================================
# CHEST PAIN
# ============================================================

b_dapeito <- subset(BANCO, !is.na(dapeito) & tepoultimo == 1)

m_dapeito <- metaprop(
  event = dapeito,
  n = dapeitot,
  studlab = autorano,
  data = b_dapeito,
  method = "Inverse",
  common = FALSE,
  prediction = TRUE,
  sortvar = b_dapeito$ano,
  print.pval = TRUE,
  digits.pval = 3
)

m_dapeito

tiff(
  "m_dapeito.tiff",
  res = 300,
  width = 30,
  height = 40,
  units = "cm",
  compression = "lzw+p"
)

meta::forest(m_dapeito)
```

```
dev.off()


# =============================================================
# PALPITATIONS
# =============================================================

b_cardio <- subset(BANCO, !is.na(cardio) & tepoultimo == 1)

m_cardio <- metaprop(
  event = cardio,
  n = cardiot,
  studlab = autorano,
  data = b_cardio,
  method = "Inverse",
  common = FALSE,
  prediction = TRUE,
  sortvar = b_cardio$ano,
  print.pval = TRUE,
  digits.pval = 3
)

m_cardio

tiff(
  "m_cardio.tiff",
  res = 300,
  width = 30,
  height = 40,
  units = "cm",
  compression = "lzw+p"
)

meta::forest(m_cardio)

dev.off()


# =============================================================
# GASTROINTESTINAL SYMPTOMS
# =============================================================

b_tgi <- subset(BANCO, !is.na(tgi) & tepoultimo == 1)

m_tgi <- metaprop(
  event = tgi,
  n = tgit,
  studlab = autorano,
  data = b_tgi,
  method = "Inverse",
  common = FALSE,
  prediction = TRUE,
  sortvar = b_tgi$ano,
  print.pval = TRUE,
  digits.pval = 3
)

m_tgi

tiff(
  "m_tgi.tiff",
  res = 300,
  width = 30,
  height = 40,
  units = "cm",
  compression = "lzw+p"
)

meta::forest(m_tgi)
```

```
dev.off()
```
