# Supplementary material for: Persistent COVID‐19 Symptoms in General Population and Healthcare Workers: Systematic Review With Meta‐Analysis
Source: Health Sci Rep. 2026 Oct 29;9(11):e73456. doi: 10.1002/hsr2.73456 (PMC13720233; doi:10.1002/hsr2.73456)
Supplement: Supplementary file 3 [file HSR2-9-e73456-s002.docx]

**Supplementary 4 - Egger’s Test and Funnel plot - Porto Alegre, RS, Brazil, 2026.**

The data below present the results of Egger’s test and the funnel plot for each outcome evaluated.

**Fatigue**

Test result: t = -2.38, df = 49, p-value = 0.0212

Bias estimate: -3.8045 (SE = 1.5975)

**Dyspnea**

Test result: t = 1.94, df = 45, p-value = 0.0589

Bias estimate: 4.6799 (SE = 2.4145)

**Arthralgia**

Test result: t = -1.04, df = 18, p-value = 0.3125

Bias estimate: -1.8200 (SE = 1.7515)

**Myalgia**

Test result: t = -2.46, df = 25, p-value = 0.0211

Bias estimate: -4.0829 (SE = 1.6585)

**Headache**

Test result: t = -0.17, df = 36, p-value = 0.8646

Bias estimate: -0.4682 (SE = 2.7251)

**Hair loss**

Test result: t = 5.63, df = 18, p-value < 0.0001

Bias estimate: 8.3280 (SE = 1.4803)

**Olfactory dysfunction**

Test result: t = -0.44, df = 28, p-value = 0.6668

Bias estimate: -0.5851 (SE = 1.3444)

**Palpitations**

Test result: t = 1.65, df = 22, p-value = 0.1138

Bias estimate: 2.9233 (SE = 1.7753)

**Cough**

Test result: t = 0.54, df = 35, p-value = 0.5945

Bias estimate: 0.9420 (SE = 1.7534)

**Dizziness/fainting**

Test result: t = 1.55, df = 21, p-value = 0.1365

Bias estimate: 2.2330 (SE = 1.4423)

**Chest pain**

Test result: t = 0.46, df = 34, p-value = 0.6504

Bias estimate: 0.5532 (SE = 1.2098)

**Taste changes**

Test result: t = -1.41, df = 26, p-value = 0.1718

Bias estimate: -1.4293 (SE = 1.0173)

**Gastrointestinal symptoms**

Test result: t = 0.95, df = 38, p-value = 0.3499

Bias estimate: 1.6168 (SE = 1.7082)

**Skin alterations**

Test result: t = 0.09, df = 19, p-value = 0.9323

Bias estimate: 0.1148 (SE = 1.3339)

**Fever/shivering**

Test result: t = -2.55, df = 15, p-value = 0.0223

Bias estimate: -2.1634 (SE = 0.8492)
