# Supplementary material for: Persistent COVID‐19 Symptoms in General Population and Healthcare Workers: Systematic Review With Meta‐Analysis
Source: Health Sci Rep. 2026 Oct 29;9(11):e73456. doi: 10.1002/hsr2.73456 (PMC13720233; doi:10.1002/hsr2.73456)
Supplement: Supplementary file 4 [file HSR2-9-e73456-s004.docx]

**Supplementary 6 – Additional information on each article included in the systematic review. Porto Alegre. RS. Brazil. 2026.**

| **Id** | **Cohort** | **Country** | **Sex** | **Mean age** | **Comorbidities** | **Followup period** | **Sample** | **Meta-analysis outcomes** |
| --- | --- | --- | --- | --- | --- | --- | --- | --- |
| González-Hermosillo 2021 | Prospective | Mexico | M: 65.4%  F: 34.6% | 51.0 | DM 31.5%; SAH 40.8%; Card 16.9%; Pulm 3.1%; Renal 10.8%; Immune 11.5%; Psyq 1.5%; Overweight 47.7%; Obesity 29.2% | 6 | 130 | Fatigue; headache; myalgia; arthralgia; olfactory dysfunctions; taste alterations; Dizziness/syncope; dyspnea; chest pain; cardio; gastrointestinal alterations |
| Hossain 2021* | Prospective | Bangladesh | M: 72.4%  F: 27.6% | 38.8 | DM 22.7%; SAH 25.6%; Card 6.0%; Pulm 6.9%; Renal 1.7% | 5 | ^‡^133 ^§^2,065 | Fatigue; headache; taste alterations; dyspnea; cough; chest pain |
| Malinowska 2021 | Prospective | Poland | M: 56.7%  F: 22.4% | 53.0 | - | 6 | 67 | Fatigue; hair loss; olfactory dysfunctions; Dizziness/syncope; dyspnea; cardio |
| Martinez 2021 | Retrospective | Switzerland | M: 24.6%  F: 75.4% | 37.0 | SAH 7.0%; Pulm 0.8%; Asthma 10.4%; Thyroid 4.7%; Psyq 1.9% | 5.5 | 260 | Fatigue; headache; myalgia; arthralgia; hair loss; Dizziness/syncope; dyspnea; cough; chest pain; cardio; gastrointestinal alterations |
| Och 2021 | Prospective | Poland | M: 49.4%  F: 50.6% | 70.0 | DM 50.6%; SAH 93.7% | 6 | 73 | Fatigue; headache; myalgia; arthralgia; fever; hair loss; skin alterations; olfactory dysfunctions; taste alterations; Dizziness/syncope; chest pain; cardio; gastrointestinal alterations |
| Scherlinger 2021 | Prospective | France | M: 40.0%  F: 60.0% | 40.0 | DM 10.0%; SAH 6.7%; Cancer 3.3%; Neuro 6.7% | 6 | 30 | Fatigue; headache; myalgia; arthralgia; fever; dyspnea; cough; chest pain; gastrointestinal alterations |
| Ali 2022 | Prospective | USA | M: 26.9%  F: 73.1% | 42.8 | - | 14.5 | 27 | Fatigue; headache; olfactory dysfunctions; taste alterations; Dizziness/syncope; dyspnea; chest pain; cardio; gastrointestinal alterations |
| Amorim 2022 | Prospective | Brazil | M: 59.4%  F: 40.6% | 48.0 | DM 25.4%; SAH 70.3%; Card 4.0%; Pulm 0.9%; Hepat 1.4%; Cancer 2.2% | 3 | 780 | Fatigue; headache; Dizziness/syncope; dyspnea; gastrointestinal alterations |
| Aparisi 2022 | Prospective | Spain | M: 35.7%  F: 64.3% | 54.8 | DM 4.3%; SAH 25.7%; Card 2.9%; Pulm 7.1% | 3 | 70 | Fatigue; headache; myalgia; olfactory dysfunctions; taste alterations; dyspnea; chest pain; cardio |
| Asadi-Pooya 2022 | Prospective | Iran | M: 54.7%  F: 45.3% | 41.0 | - | 3 | 2,696 | Fatigue; myalgia; olfactory dysfunctions; dyspnea; cough; chest pain |
| Buonsenso 2022 | Prospective | Italy | M: 54.3%  F: 45.7% | 44.6 | - | 7.5 | 107 | Skin alterations; gastrointestinal alterations |
| Carazo 2022 | Retrospective | Canada | M: 22.8%  F: 77.2% | 40.0 | - | 3 | 1,783 | Fatigue; headache; dyspnea; cough; chest pain; gastrointestinal alterations |
| Desgranges 2022* | Prospective | Switzerland | ^‡^M: 30.6% F: 69.4% ^§^M: 51.8% F: 48.2% | ^‡^38.0 ^§^51.0 | aDM 2.5%; SAH 8.9%; Card 1.1%; Cancer 1.4%; Asthma 7.5%; Overweight 39.0%; Obesity 15.0%  bDM 11.0%; SAH 29.0%; Card 5.1%; Cancer 8.0%; Asthma 16.0%; Overweight 58.0%; Obesity 23.0% | 5 | ^‡^281 ^§^137 | Fatigue; headache; hair loss; dyspnea; cough; chest pain; gastrointestinal alterations |
| Dryden 2022 | Prospective | South Africa | M: 48.7%  F: 51.3% | 52.0 | DM 22.3%; SAH 35.7%; Card 5.9%; Pulm 1.3%; Renal 1.6%; Cancer 1.4%; HIV: 5.1%; Asthma 4.9%; Osteomusc 1.4%; Thyroid 1.3%; Dyslip 5.6%; Psyq 1.1%; Obesity 25.3% | 3 | 1,873 | Fatigue; headache; myalgia; arthralgia; fever; skin alterations; olfactory dysfunctions; taste alterations; Dizziness/syncope; dyspnea; cough; chest pain; gastrointestinal alterations |
| Estrada-Codecido 2022 | Retrospective | Canada | M: 44.6%  F: 55.4% | 38.0 | DM 9.2%; SAH 21.9%; Card 7.1%; Pulm 2.6%; Renal 3.0%; Hepat 1.3%; Cancer 7.1%; HIV 0.4%; Asthma 10.5%; Neuro 5.4%; Osteomusc 2.6%; Blood 2.1%; Obesity 7.9% | 3 | 466 | Fatigue; headache; myalgia; arthralgia; fever; hair loss; skin alterations; olfactory dysfunctions; taste alterations; dyspnea; cough; chest pain; gastrointestinal alterations |
| Fang 2022 | Prospective | China | M: 47.9%  F: 52.1% | 68.0 | DM 19.0%; SAH 41.8%; Card 24.1%; Pulm 1.5%; Renal 2.5%; Hepat 4.3%; Cancer 2.6%; Neuro 5.8% | 12 | 1,233 | Fatigue; headache; myalgia; fever; olfactory dysfunctions; taste alterations; Dizziness/syncope; dyspnea; cough; chest pain; cardio; gastrointestinal alterations |
| Fernández-de-Las-Peñas 2022 | Prospective | Spain | M: 53.5%  F: 46.5% | 61.0 | DM 12.0%; SAH 26.1%; Card 11.9%; Pulm 3.9%; Asthma 6.4%; Obesity 4.5% | 8.4 | 1,969 | Fatigue; skin alterations; olfactory dysfunctions; taste alterations; dyspnea; cardio; gastrointestinal alterations |
| Förster 2022 | Retrospective | Germany | M: 43.5%  F: 56.5% | 53.0 | DM 6.9%; SAH 27.1%; Card 12.7%; Pulm 11.8%; Renal 3.0%; Hepat 1.2%; Gastro 11.7%; Cancer 5.1%; Osteomusc 21.7%; Alergia 2.2%; Blood 4.3%; Thyroid 4.2%; Psyq 9.1% | 7.8 | 1,459 | Fatigue; headache; fever; hair loss; skin alterations; olfactory dysfunctions; taste alterations; Dizziness/syncope; dyspnea; cough; cardio; gastrointestinal alterations |
| Freire 2022 | Prospective | Brazil | M: 53.2%  F: 46.8% | 56.0 | DM 36.0%; SAH 57.3%; Card 12.5%; Pulm 5.4%; Renal 12.9%; Hepat 5.7%; Cancer 5.0%; Asthma 3.6% | 8.5 | 822 | Myalgia; cough; chest pain; gastrointestinal alterations |
| González 2022 | Prospective | Spain | M: 64.9% F: 35.1% | 61.0 | DM 19.0%; SAH 43.8%; Card 9.5%; Pulm 7.6%; Renal 3.8%; Asthma 7.6%; Obesity 41.9% | 12 | 97 | Fatigue; headache; arthralgia; hair loss; skin alterations; Dizziness/syncope; dyspnea; chest pain; cardio; gastrointestinal alterations |
| Hastie 2022 | Prospective | Scotland | M: 36.4%  F: 63.6% | 44.0 | DM 4.3%; SAH 0.9%; Card 2.7%; Pulm 21.5%; Renal 0.3%. Hepat 0.5%; Cancer 1.0%; HIV 0.1%; Neuro 2.2%; Osteomusc 5.5%; Embolia 0.2%; Psyq 42.5%; Overweight 7.9% | 12 | 33,281 | Fatigue; headache; myalgia; arthralgia; skin alterations; olfactory dysfunctions; taste alterations; Dizziness/syncope; dyspnea; cough; chest pain; cardio; gastrointestinal alterations |
| Kalak 2022 | Prospective | Israel | M: 50.0%  F: 50.0% | 52.1 | DM 16.9%; SAH 23.5%; Card 9.0%; Pulm 1.8%; Renal 2.4%; Gastro 0.6%; Cancer 1.8%; Immune 1.8%; Asthma 6.6% | 18 | 166 | Fatigue; olfactory dysfunctions; taste alterations; dyspnea; cough; chest pain |
| Marasco 2022 | Prospective | Italy | M: 59.3%  F: 40.7% | 49.9 | DM 14.5%; Card 28.2%; Pulm 6.5%; Renal 4.6%; Hepat 3.1%; Cancer 2.1%; Immune 2.8%; Neuro 2.6%; Osteomusc 2.6%; Alergia 2.9%; Blood 1.1%; Psyq 1.0% | 12 | 435 | Gastrointestinal alterations |
| Nguyen 2022 | Prospective | France | M: 36.2%  F: 63.8% | 40.0 | DM 5.0%; SAH 10.7%; Card 2.1%; Pulm 10.2%; Cancer 2.0%; Obesity 10.2% | 6 | 605 | Olfactory dysfunctions; taste alterations |
| Pérez-González 2022 | Prospective | Spain | M: 59.7%  F: 40.3% | 57.0 | DM 10.5%; SAH 26.2%; Card 8.5%; Pulm 6.0%; Renal 1.2%; Hepat 0.8%; Cancer 2.0%; HIV 1.2%; Asthma 8.5%; Obesity 30.2% | 6 | 248 | Fatigue; headache; myalgia; arthralgia; hair loss; olfactory dysfunctions; taste alterations; dyspnea; cough; chest pain; gastrointestinal alterations |
| Petersen 2022 | Prospective | Denmark | M: 47.8%  F: 52.2% | 31.3 | - | 6 | 170 | Fatigue; headache; myalgia; arthralgia; fever; skin alterations; olfactory dysfunctions; taste alterations; dyspnea; cough; chest pain; gastrointestinal alterations |
| Rigoni 2022 | Prospective | Italy | M: 63.9%  F: 36.1% | 71.0 | DM 17.4%; SAH 61.4%; Pulm 12.1%; Renal 10.6%; Gastro15.5%; Cancer 11.9%; Immune 13.2%; Obesity 11.7% | 12 | 344 | Myalgia; arthralgia; hair loss; skin alterations; olfactory dysfunctions; taste alterations; Dizziness/syncope; dyspnea; cardio; gastrointestinal alterations |
| Rivera-Izquierdo 2022 | Prospective | Spain | M: 57.4%  F: 42.6% | 61.2 | - | 12 | 453 | Fatigue; headache; skin alterations; dyspnea; chest pain; gastrointestinal alterations |
| Seeßle 2022 | Prospective | Germany | M: 44.8%  F: 55.2% | 57.0 | DM 7.3%; SAH 35.1%; Card 4.2%; Cancer 4.2%; Immune 5.2%; Asthma 12.5%; Psyq 7.3%; Obesity 24.0% | 12 | 96 | Fatigue; headache; fever; hair loss; olfactory dysfunctions; taste alterations; Dizziness/syncope; dyspnea; cough; cardio; gastrointestinal alterations |
| Strahm 2022 | Prospective | Switzerland | M: 17.8%  F: 82.2% | 38.9 | - | 4 | 556 | Fatigue; headache; myalgia; arthralgia; hair loss; Dizziness/syncope; dyspnea; cough; chest pain; cardio |
| Sun 2022 | Retrospective | China | M: 44.8%  F: 55.2% | 62.0 | DM 15.9%; SAH 37.1%; Card 13.3%; Pulm 4.1%; Renal 1.3%; Hepat 4.5%; Cancer 4.3%; Asthma 0.4% | 15 | 534 | Fatigue; headache; myalgia; arthralgia; fever; hair loss; olfactory dysfunctions; taste alterations; Dizziness/syncope; dyspnea; cough; chest pain; cardio; gastrointestinal alterations |
| Takakura 2022 | Retrospective | Japan | M: 47.9%  F: 52.1% | 35.8 | - | 3 | 257 | Fatigue; fever; hair loss; olfactory dysfunctions; Dizziness/syncope; cardio; gastrointestinal alterations |
| Tartof 2022 | Retrospective | USA | M: 46.3%  F: 53.7% | 41.2 | DM 13.2%; SAH 17.9%; Card 5.0%; Pulm 1.6%; Renal 3.0%; Cancer 1.9%; Immune 1.3%; Asthma 0.8%; Neuro 0.9%; Blood 0.1%; Overweight 18.4% | 5 | 127,859 | Headache; hair loss; skin alterations; dyspnea; cough; cardio; gastrointestinal alterations |
| Titze-de-Almeida 2022 | Prospective | Brazil | M: 39.0%  F: 61.0% | 41.2 | DM 8.9%; SAH 18.6%; Card 2.5%; Pulm 7.2%; Renal 3.4%; Cancer 1.3%; Immune 3.0% | 6.5 | 236 | Fatigue; headache; myalgia; fever; olfactory dysfunctions; taste alterations; dyspnea; cough; gastrointestinal alterations |
| Visconti 2022 | Prospective | Brazil | M: 47.7%  F: 52.3% | 60.5 | DM 31.8%; SAH 73.9%; Card 21.6%; Pulm 8.0%; Hepat 9.1%; Cancer 13.6%; Immune 8.0%; Asthma 5.7%; Obesity 20.5% | 12 | 88 | Fatigue; headache; myalgia; arthralgia; dyspnea; cough; chest pain |
| Wisnivesky 2022 | Prospective | USA | M: 35.1%  F: 64.9% | 50.1 | DM 11.7%; SAH 32.2%; Card 4.0%; Pulm 3.3%; Cancer 11.5%; Asthma 27.8%; Overweight 29.4%; Obesity 34.0% | 6 | 453 | Fatigue; olfactory dysfunctions; dyspnea; cough |
| Zuschlag 2022 | Retrospective | Germany | M: 54.3%  F: 45.7% | 62.0 | - | 12 | 162 | Fatigue; headache; skin alterations; Dizziness/syncope; dyspnea; cough; chest pain; gastrointestinal alterations |
| Ballouz 2023 | Prospective | Switzerland | M: 50.4%  F: 49.6% | 50.0 | DM 2.8%; SAH 15.1%; Card 5.9%; Pulm 8.7%; Hepat 0.8%; Cancer 5.8%; Immune 0.3% | 6 | 504 | Fatigue; myalgia; arthralgia; fever; hair loss; skin alterations; Dizziness/syncope; dyspnea; cough; chest pain; cardio; gastrointestinal alterations |
| Bamps 2023^†^ | Prospective | Switzerland | M: 44.51%  F: 55.49% | 55.0 | DM 11.2%; SAH 28.9%; Card 14.1%; Pulm 3.8%; Renal 4.0%; Hepat 0.4%; Cancer 4.9%; Asthma 7.4%; Overweight 32.3%; Obesity 15.8% | 12 | 474 | ** |
| Benoit-Piau 2023 | Prospective | Canada | M: 38.7%  F: 61.3% | 46.6 | Card 19.3%; Pulm 13.1%; Cancer 2.3%; Osteomusc 19.6%; Psyq 20.5% | 6 | 1,349 | Gastrointestinal alterations |
| Brunvoll 2023 | Prospective | Norway | M: 28.7%  F: 71.3% | 48.0 | - | 9 | 1,420 | Fatigue; dyspnea |
| Cecchetto 2023 | Retrospective | Italy | M: 60.9%  F: 39.1% | 65.0 | DM 15.1%; SAH 44.9%; Card 5.3%; Renal 3.1%; Dyslip 14.7%; Obesity 25.8% | 5 | 225 | Dyspnea |
| D'Ávila 2023 | Prospective | Brazil | M: 19.4%  F: 80.6% | 42.2 | - | 6 | 174 | Fatigue; dyspnea; cough |
| Gentilotti 2023 | Prospective | France. Italy. Argentina. Spain. Netherlands | M: 56.6%  F: 43.4% | 57.2 | DM 8.6%; Card 39.5%; Pulm 16.5%; Renal: 5.0%; Hepat 2.9%; Cancer 6.7%; Immune 5.3%; HIV 2.1% | 12 | 1,796 | Fatigue; headache; myalgia; arthralgia; fever; skin alterations; olfactory dysfunctions; taste alterations; Dizziness/syncope; dyspnea; cough; chest pain; gastrointestinal alterations |
| Grewal 2023 | Prospective | Canada | M: 61.8%  F: 38.2% | 63.0 | DM 23.7%; SAH 34.2%; Card 18.4%; Pulm 21.1%; Renal 9.2%; Cancer 10.5%; Dyslip 31.6% | 12 | 76 | Dyspnea |
| Hyassat 2023 | Prospective | Jordan | M: 29.3%  F: 70.7% | 34.4 | DM 5.7%; SAH 2.1%; Card 1.4%; Pulm 0.7%; Neuro 0.7%; Alergia 6.4%; Overweight 39.3%. Obesity 12.1% | 12 | 140 | Fatigue; headache; fever; skin alterations; olfactory dysfunctions; taste alterations; Dizziness/syncope; dyspnea; cough; chest pain; cardio; gastrointestinal alterations |
| Klinkhammer 2023 | Prospective | Netherlands | M: 69.8%  F: 30.2% | 64.0 | DM 13.7%; SAH 33.3%; Card 20.8%; Pulm 9.3%; Renal 5.5%; Cancer 2.2%; Neuro 7.7% | 9 | 205 | Fatigue |
| Krishnadath 2023 | Prospective | Suriname | M: 37.7%  F: 62.3% | 49.0 | DM 24.5%; SAH 26.4%; Card 13.2%; Pulm 6.6%; Renal 1.9%; Neuro 6.6% | 3.5 | 106 | Fatigue; headache; myalgia; arthralgia; fever; hair loss; olfactory dysfunctions; taste alterations; dyspnea; cough; chest pain; cardio; gastrointestinal alterations |
| Lemhöfer 2023 | Prospective | Germany | M: 33.6%  F: 66.4% | 49.5 | Card 4.4%; Pulm 14.4%; Embolia 3.0%; Psyq 13.7% | 9.6 | 952 | Fatigue; skin alterations; chest pain; gastrointestinal alterations |
| Magel 2023 | Prospective | Canada | M: 63.6%  F: 36.4% | 61.1 | SAH 22.7%; Pulm 52.3%; Renal 19.3%; Hepat 11.4%; Gastro 4.5%; Cancer 13.6%; Immune 9.1%; HIV 10.2% | 6 | 79 | Fatigue |
| Monteiro 2023 | Prospective | Brazil | M: 30.2%  F: 69.8% | 41.4 | - | 9 | 48 | Fatigue; headache; olfactory dysfunctions; taste alterations; Dizziness/syncope; cardio; gastrointestinal alterations |
| Oliveira 2023 | Retrospective | Portugal | M: 40.8%  F: 59.2% | 46.0 | DM 6.3%; SAH 22.5%; Card 5.3%; Pulm 1.8%; Renal 1.4%; Cancer 6.3%; Immune 5.6%; HIV 0.4%; Asthma 6.7%; Neuro 1.8%; Psyq 5.3% | 3 | 284 | Fatigue; skin alterations; gastrointestinal alterations |
| Peghin 2023 | Prospective | Italy | M: 46.5%  F: 53.5% | 54.7 | DM 6.5%; SAH 20.4%; Card 1.7%; Pulm 3.5%; Hepat 3.0%; Psyq 1.3%; Obesity 12.6% | 24 | 230 | Fatigue; headache; hair loss; skin alterations; dyspnea; cough; chest pain; gastrointestinal alterations |
| Ranucci 2023 | Prospective | Italy | M: 66.1%  F: 33.9% | 63.1 | DM 16.5%; SAH 45.5%; Card 33.1%; Pulm 5.0%; Renal 6.6%; Hepat 3.3%; Cancer 9.1%; Neuro 5.0%; Psyq 27.3%; Obesity 23.1% | 17 | 121 | Fatigue; headache; myalgia; arthralgia; fever; olfactory dysfunctions; taste alterations; dyspnea; cough; chest pain; cardio |
| Serrano 2023 | Prospective | Colombia | M: 70.4%  F: 29.6% | 62.0 | DM 14.1%; SAH 36.3%; Card 3.0% | 12 | 135 | Fatigue; headache; myalgia; dyspnea; cough |
| Silva 2023 | Prospective | Brazil | M: 36.1%  F: 63.9% | 38.4 | DM 4.1%; SAH 38.8%; Card 9.5%; Renal 5.4%; Hepat 0.7%; HIV 0.7%; Asthma 23.1% | 12 | 69 | Fatigue; headache; myalgia; hair loss; olfactory dysfunctions; taste alterations; cough |
| Smith 2023 | Prospective | Belgium | M: 35.4%  F: 64.6% | 43.1 | - | 3 | 5,727 | Fatigue; headache; myalgia; arthralgia; skin alterations; Dizziness/syncope; dyspnea; cough; chest pain; cardio; gastrointestinal alterations |
| Steinmetz 2023 | Prospective | Germany | M: 21.5%  F: 78.5% | 48.2 | DM 8.2%; SAH 28.5%; Pulm 10.1%; Neuro 7.6%; Osteomusc 26.6%; Psyq 11.9% | 6 | 104 | Fatigue; headache; myalgia; arthralgia; fever; hair loss; olfactory dysfunctions; taste alterations; Dizziness/syncope; dyspnea; cough; chest pain; cardio; gastrointestinal alterations |
| Sykes 2023 | Retrospective | England | M: 62.5%  F: 37.5% | 62.0 | DM 26.3%; SAH 42.4%; Card 2.8%; Pulm 13.9%; Renal 2.8%; Asthma 19.4%; Embolism3.5% | 3 | 144 | Fatigue; myalgia; skin alterations; olfactory dysfunctions; taste alterations; dyspnea; cough; chest pain |
| * Studies with general population and healthcare professionals. stratified analysis.\| ^†^ Study included outcome which was not used in the meta-analysis. | | | | | | | | |
| ^‡^ healthcare professionals' data \| ^§^ general population's data.  DM: diabetes mellitus. SAH: systemic arterial hypertension. Card: cardiac diseases. Pulm; pulmonary diseases. Renal: renal diseases. Hepat: hepatological diseases. Neuro: neurologic diseases. Gastro: gastrointestinal diseases. Osteomusc: osteomuscular diseases. Thyroid; thyroid diseases. Dyslip: dyslipidemia. Blood: hematological (ex: anemia). Immuno: immunological. Psyq: psychiatric diseases. | | | | | | | | |
