# Supplementary material for: Persistent COVID‐19 Symptoms in General Population and Healthcare Workers: Systematic Review With Meta‐Analysis
Source: Health Sci Rep. 2026 Oct 29;9(11):e73456. doi: 10.1002/hsr2.73456 (PMC13720233; doi:10.1002/hsr2.73456)
Supplement: Supplementary file 5 [file HSR2-9-e73456-s006.docx]

**Supplementary 7 – Forest plots other symptoms. Porto Alegre, RS, Brazil, 2026.**

**Dyspnea**

**Arthralgia**

**Myalgia**

**Headache**

**Hair loss**

**Olfactory dysfunction**

**Palpitations**

**Cough**

**Dizziness/fainting**

**Chest pain**

**Taste changes**

**Gastrointestinal symptoms**

**Skin alterations**

**Fever/shivering**
