# Supplementary material for: Persistent COVID‐19 Symptoms in General Population and Healthcare Workers: Systematic Review With Meta‐Analysis
Source: Health Sci Rep. 2026 Oct 29;9(11):e73456. doi: 10.1002/hsr2.73456 (PMC13720233; doi:10.1002/hsr2.73456)
Supplement: Supplementary file 6 [file HSR2-9-e73456-s007.docx]

**Supplementary 8 – Articles reporting each symptom listed for meta-analysis in the systematic review. Porto Alegre, RS, Brazil, 2026**

| **Outcome** | **Nº** | **Identification of the articles which outcomes are mentioned** | | | | |
| --- | --- | --- | --- | --- | --- | --- |
| FATIGUE | 49 | González-Hermosillo 2021 Hossain 2021 Malinowska 2021 Martinez 2021 Och 2021 Scherlinger 2021 Ali 2022 Amorim 2022 Aparisi 2022 Asadi-Pooya 2022 | Carazo 2022 Desgranges 2022 Dryden 2022 Estrada-Codecido 2022 Fang 2022 Fernández-de-Las-Peñas 2022 Förster 2022 González 2022 Hastie 2022 Kalak 2022 | Pérez-González 2022  Petersen 2022  Rivera-Izquierdo 2022  Seeßle 2022  Strahm 2022  Sun 2022  Takakura 2022  Titze-de-Almeida 2022  Visconti 2022  Wisnivesky 2022 | Zuschlag 2022  Ballouz 2023  Brunvoll 2023  D'Ávila 2023  Gentilotti 2023  Hyassat 2023  Klinkhammer 2023  Krishnadath 2023  Lemhöfer 2023  Magel 2023 | Monteiro 2023  Oliveira 2023  Peghin 2023  Ranucci 2023  Serrano 2023  Silva 2023  Smith 2023  Steinmetz 2023  Sykes 2023 |
| DYSPNEA | 45 | González-Hermosillo 2021 Hossain 2021 Malinowska 2021 Martinez 2021 Scherlinger 2021 Ali 2022 Amorim 2022 Aparisi 2022 Asadi-Pooya 2022 Carazo 2022 | Desgranges 2022 Dryden 2022 Estrada-Codecido 2022 Fang 2022 Fernández-de-Las-Peñas 2022 Förster 2022 González 2022 Hastie 2022 Kalak 2022 Pérez-González 2022 | Petersen 2022 Rigoni 2022 Rivera-Izquierdo 2022 Seeßle 2022 Strahm 2022 Sun 2022  Tartof 2022  Titze-de-Almeida 2022  Visconti 2022  Wisnivesky 2022 | Zuschlag 2022 Ballouz 2023 Brunvoll 2023 Cecchetto 2023 D'Ávila 2023 Gentilotti 2023  Grewal 2023  Hyassat 2023  Krishnadath 2023  Peghin 2023 | Ranucci 2023 Serrano 2023 Smith 2023 Steinmetz 2023 Sykes 2023 |
| ARTHRALGIA | 20 | González-Hermosillo 2021 Martinez 2021 Och 2021 Scherlinger 2021 Dryden 2022 Estrada-Codecido 2022 González 2022 Hastie 2022 Pérez-González 2022 Petersen 2022 | Rigoni 2022  Strahm 2022  Sun 2022  Visconti 2022  Ballouz 2023  Gentilotti 2023  Krishnadath 2023  Ranucci 2023  Smith 2023  Steinmetz 2023 |  |  |  |
| MYALGIA | 27 | González-Hermosillo 2021 Martinez 2021 Och 2021 Scherlinger 2021 Aparisi 2022 Asadi-Pooya 2022 Dryden 2022 Estrada-Codecido 2022 Fang 2022 Freire 2022 | Hastie 2022  Pérez-González 2022  Petersen 2022  Rigoni 2022  Strahm 2022  Sun 2022  Titze-de-Almeida 2022  Visconti 2022  Ballouz 2023  Gentilotti 2023 | Krishnadath 2023  Ranucci 2023  Serrano 2023  Silva 2023  Smith 2023  Steinmetz 2023  Sykes 2023 |  |  |
| HEADACHE | 36 | González-Hermosillo 2021 Hossain 2021 Martinez 2021 Och 2021 Scherlinger 2021 Ali 2022 Amorim 2022 Aparisi 2022 Carazo 2022 Desgranges 2022 | Dryden 2022  Estrada-Codecido 2022  Fang 2022  Förster 2022  González 2022  Hastie 2022  Pérez-González 2022  Petersen 2022  Rivera-Izquierdo 2022  Seeßle 2022 | Strahm 2022  Sun 2022  Tartof 2022  Titze-de-Almeida 2022  Visconti 2022  Zuschlag 2022  Gentilotti 2023  Hyassat 2023  Krishnadath 2023  Monteiro 2023 | Peghin 2023  Ranucci 2023  Serrano 2023  Silva 2023  Smith 2023  Steinmetz 2023 |  |
| HAIR LOSS | 19 | Malinowska 2021 Martinez 2021 Och 2021 Desgranges 2022 Estrada-Codecido 2022 Förster 2022 González 2022 Pérez-González 2022 Rigoni 2022 Seeßle 2022 | Strahm 2022  Sun 2022  Takakura 2022  Tartof 2022  Ballouz 2023  Krishnadath 2023  Peghin 2023  Silva 2023  Steinmetz 2023 |  |  |  |
| OLFACTORY DYSFUNCTIONS | 30 | González-Hermosillo 2021 Malinowska 2021 Och 2021 Ali 2022 Aparisi 2022 Asadi-Pooya 2022 Dryden 2022 Estrada-Codecido 2022 Fang 2022 Fernández-de-Las-Peñas 2022 | Förster 2022  Hastie 2022  Kalak 2022  Nguyen 2022  Pérez-González 2022  Petersen 2022  Rigoni 2022  Seeßle 2022  Sun 2022  Takakura 2022 | Titze-de-Almeida 2022  Wisnivesky 2022  Gentilotti 2023  Hyassat 2023  Krishnadath 2023  Monteiro 2023  Ranucci 2023  Silva 2023  Steinmetz 2023  Sykes 2023 |  |  |
| PALPITATIONS | 24 | González-Hermosillo 2021 Malinowska 2021 Martinez 2021 Och 2021 Ali 2022 Aparisi 2022 Fang 2022 Fernández-de-Las-Peñas 2022 Förster 2022 González 2022 | Hastie 2022 Rigoni 2022 Seeßle 2022 Strahm 2022 Sun 2022 Takakura 2022 Tartof 2022 Ballouz 2023 Hyassat 2023 Krishnadath 2023 | Monteiro 2023  Ranucci 2023  Smith 2023  Steinmetz 2023 |  |  |
| COUGH | 35 | Hossain 2021 Martinez 2021 Scherlinger 2021 Asadi-Pooya 2022 Carazo 2022 Desgranges 2022 Dryden 2022 Estrada-Codecido 2022 Fang 2022 Förster 2022 | Freire 2022 Hastie 2022 Kalak 2022 Pérez-González 2022 Petersen 2022 Seeßle 2022 Strahm 2022 Sun 2022 Tartof 2022 Titze-de-Almeida 2022 | Visconti 2022 Wisnivesky 2022 Zuschlag 2022 Ballouz 2023 D'Ávila 2023 Gentilotti 2023 Hyassat 2023 Krishnadath 2023 Peghin 2023 Ranucci 2023 | Serrano 2023  Silva 2023  Smith 2023  Steinmetz 2023  Sykes 2023 |  |
| DIZZINESS/SYNCOPE | 23 | González-Hermosillo 2021 Malinowska 2021 Martinez 2021 Och 2021 Ali 2022 Amorim 2022 Dryden 2022 Fang 2022 Förster 2022 González 2022 | Hastie 2022  Rigoni 2022  Seeßle 2022  Strahm 2022  Sun 2022  Takakura 2022  Zuschlag 2022  Ballouz 2023  Gentilotti 2023  Hyassat 2023 | Monteiro 2023  Smith 2023  Steinmetz 2023 |  |  |
| CHEST PAIN | 34 | González-Hermosillo 2021 Hossain 2021 Martinez 2021 Och 2021 Scherlinger 2021 Ali 2022 Aparisi 2022 Asadi-Pooya 2022 Carazo 2022 Desgranges 2022 | Dryden 2022  Estrada-Codecido 2022  Fang 2022  Freire 2022  González 2022  Hastie 2022  Kalak 2022  Pérez-González 2022  Petersen 2022  Rivera-Izquierdo 2022 | Strahm 2022  Sun 2022  Visconti 2022  Zuschlag 2022  Ballouz 2023  Gentilotti 2023  Hyassat 2023  Krishnadath 2023  Lemhöfer 2023  Peghin 2023 | Ranucci 2023  Smith 2023  Steinmetz 2023  Sykes 2023 |  |
| TASTE ALTERATIONS | 27 | González-Hermosillo 2021 Hossain 2021 Och 2021 Ali 2022 Aparisi 2022 Dryden 2022 Estrada-Codecido 2022 Fang 2022 Fernández-de-Las-Peñas 2022 Förster 2022 | Hastie 2022 Kalak 2022 Nguyen 2022 Pérez-González 2022 Petersen 2022 Rigoni 2022 Seeßle 2022 Sun 2022 Titze-de-Almeida 2022 Gentilotti 2023 | Hyassat 2023  Krishnadath 2023  Monteiro 2023  Ranucci 2023  Silva 2023  Steinmetz 2023  Sykes 2023 |  |  |
| GASTROINTESTINAL ALTERATIONS | 39 | González-Hermosillo 2021 Martinez 2021 Och 2021 Scherlinger 2021 Ali 2022 Amorim 2022 Buonsenso 2022 Carazo 2022 Desgranges 2022 Dryden 2022 | Estrada-Codecido 2022  Fang 2022  Fernández-de-Las-Peñas 2022  Förster 2022  Freire 2022  González 2022  Hastie 2022  Marasco 2022  Pérez-González 2022  Petersen 2022 | Rigoni 2022  Rivera-Izquierdo 2022  Seeßle 2022  Sun 2022  Takakura 2022  Tartof 2022  Titze-de-Almeida 2022  Zuschlag 2022  Ballouz 2023  Benoit-Piau 2023 | Gentilotti 2023  Hyassat 2023  Krishnadath 2023  Lemhöfer 2023  Monteiro 2023  Oliveira 2023  Peghin 2023  Smith 2023  Steinmetz 2023 |  |
| SKIN ALTERATIONS | 21 | Och 2021 Buonsenso 2022 Dryden 2022 Estrada-Codecido 2022 Fernández-de-Las-Peñas 2022 Förster 2022 González 2022 Hastie 2022 Petersen 2022 Rigoni 2022 | Rivera-Izquierdo 2022 Tartof 2022 Zuschlag 2022 Ballouz 2023 Gentilotti 2023 Hyassat 2023 Lemhöfer 2023 Oliveira 2023 Peghin 2023 Smith 2023 Sykes 2023 |  |  |  |
| FEVER | 17 | Och 2021 Scherlinger 2021 Dryden 2022 Estrada-Codecido 2022 Fang 2022 Förster 2022 Petersen 2022 Seeßle 2022 Sun 2022 Takakura 2022 | Titze-de-Almeida 2022  Ballouz 2023  Gentilotti 2023  Hyassat 2023  Krishnadath 2023  Ranucci 2023  Steinmetz 2023 |  |  |  |
