# Supplementary material for: Persistent COVID‐19 Symptoms in General Population and Healthcare Workers: Systematic Review With Meta‐Analysis
Source: Health Sci Rep. 2026 Oct 29;9(11):e73456. doi: 10.1002/hsr2.73456 (PMC13720233; doi:10.1002/hsr2.73456)
Supplement: Supplementary file 8 [file HSR2-9-e73456-s009.docx]

**Supplementary 9 – Subgroup analysis conducted due to high heterogeneity. Porto Alegre, RS, Brazil, 2026.**

Subgroup analysis conducted due to high heterogeneity, part 1. Porto Alegre, RS, Brazil, 2026.

Subgroup analysis conducted due to high heterogeneity, part 2. Porto Alegre, RS, Brazil, 2026.

Subgroup analysis conducted due to high heterogeneity, part 3. Porto Alegre, RS, Brazil, 2026.

Subgroup analysis conducted due to high heterogeneity, part 4. Porto Alegre, RS, Brazil, 2026.
