# Supplementary material for: Persistent COVID‐19 Symptoms in General Population and Healthcare Workers: Systematic Review With Meta‐Analysis
Source: Health Sci Rep. 2026 Oct 29;9(11):e73456. doi: 10.1002/hsr2.73456 (PMC13720233; doi:10.1002/hsr2.73456)
Supplement: Supplementary file 9 [file HSR2-9-e73456-s008.docx]

**Supplementary 5 – Complete Newcastle-Ottawa Scale by article. Porto Alegre, RS, Brazil, 2026.**
